# Rethinking governance of synthetic cells

**DOI:** 10.3389/fbioe.2026.1872353

**Published:** 2026-07-09

**Authors:** David R. Gillum, Peter A. Carr, India Hook-Barnard, Trisha Tucholski

**Affiliations:** 1 Compliance and Research Administration, University of Nevada, Reno, Reno, NV, United States; 2 RTX BBN Technologies, Cambridge, MA, United States; 3 Engineering Biology Research Consortium, Emeryville, CA, United States; 4 National Academies of Sciences, Engineering, and Medicine, Washington, DC, United States

**Keywords:** biosafety, biosecurity, biotechnology governance, synthetic cells, synthetic nucleic acids

## Abstract

Synthetic cells represent a class of engineered biological systems that span a continuum from non-replicating biochemical assemblies to genome-containing entities, exposing limitations in conventional approaches to biotechnology governance. These systems are enabled by advances in synthetic nucleic acid technologies, which provide the basis for their design, function, and programmability and expand the accessible design space of engineered biological systems. Building on a recent National Academies report, this perspective argues that continued advances in nucleic acid synthesis and design are a key driver of emerging biosafety and biosecurity challenges, particularly by challenging assumptions embedded in sequence- and organism-based approaches to oversight. We treat synthetic cells as a boundary case that highlights limitations in nucleic-acid-enabled biotechnology governance. Drawing on the report’s property-based framework, we examine how governance misalignment arises in practice through the interaction of categorical triggers, institutional boundaries, and the timing of oversight across the research and development lifecycle. We further highlight limitations in how current risk assessment approaches are applied and provide structured questions that more explicitly support the evaluation of benefits alongside risks to improve consistency in function- and context-based assessment across institutional and policy settings. Together, these observations underscore the need to align governance approaches with system properties, intended use, and deployment context as engineering biology continues to expand beyond conventional biological categories.

## 1 Introduction

Synthetic cells are an emerging area of engineering biology that involve building encapsulated cell-like systems from biomolecular components (Noireaux and Liu, 2020; Elani, 2021; Gaut and Adamala, 2021; Rothschild et al., 2024). As a research platform, they provide new ways to investigate fundamental questions about living systems while enabling controlled and modular engineering of biological functions (Adamala et al., 2017; Buddingh and van Hest, 2017; Xie and Fussenegger, 2018; Groaz et al., 2020; Rothschild et al., 2024). Synthetic cells may support applications across medicine, agriculture, environmental remediation, and manufacturing, particularly where precise, programmable biological activity is required (Khalil and Collins, 2010; Brooks and Alper, 2021; Sato et al., 2021; Rothschild et al., 2024).

Synthetic cells frequently depend on synthetic nucleic acids for their operating instructions, whether used as templates for gene expression in cell-free systems or as components of fully synthetic genomes (Hutchison et al., 2016; Noireaux and Liu, 2020; Silverman et al., 2020; Rothschild et al., 2024). Advances in synthetic nucleic acid technologies, including the ability to synthesize, modify, and deploy non-canonical or recoded genetic systems, are expanding the functional design space of synthetic cells (Pinheiro et al., 2012; Lajoie et al., 2013; Ostrov et al., 2016; Chin, 2017; Hoshika et al., 2019). In parallel, advances in artificial intelligence–enabled biodesign are further expanding this design space while complicating biosecurity approaches that rely on comparison to known sequences (Wittman et al., 2025). Together, these developments challenge assumptions embedded in existing biosecurity and biosafety frameworks, which often rely on recognizable sequences, known organisms, and established genotype–phenotype relationships (Millett et al., 2023; NRC, 2010; NRC, 2004a; NASEM, 2018; Diggans and LeProust, 2019).

The diversity of synthetic cell designs and potential uses makes them a useful test case for biotechnology governance (Zhang et al., 2011; Stemerding et al., 2019; Li et al., 2021). A recent National Academies report, *Supporting Responsible Innovation of Synthetic Cells: Biosafety, Biosecurity, and Environmental Considerations* (hereafter “the report”), finds that many risks associated with synthetic cells are comparable to those posed by chemicals, conventional engineered organisms, or naturally occurring biological systems (NASEM, 2026). At the same time, novel combinations of features, boundary-blurring architectures, and emerging deployment contexts introduce forms of uncertainty that can complicate risk–benefit assessment and governance (NASEM, 2026). And some synthetic cells—such as those with novel genetic codes, unconventional nucleotides, or even hypothetical mirror bacteria—present potential risks that are uncharted territory (Adamala et al., 2024; Evans et al., 2025; Peplow, 2025).

This paper builds on these observations by arguing that advances in synthetic nucleic acid systems are a key driver of emerging governance challenges. Synthetic cells are treated here as a boundary case for nucleic-acid-enabled biotechnology governance, sitting at the interface of chemical and biological systems and exposing limitations in how current oversight frameworks identify and manage risk. The paper makes three contributions. First, it shows how non-canonical and engineered genetic systems strain assumptions underlying sequence-based governance approaches, particularly those used for screening, attribution, and detection. Second, it builds on the report’s property-based framework by examining how governance misalignment arises in practice through the interaction of categorical triggers, institutional boundaries, and the timing of oversight across the research and development lifecycle. Third, it highlights limitations in how current risk assessment approaches are applied in practice and provides a structured set of questions that more explicitly support the evaluation of benefits alongside risks, applicable across institutional and policy settings.

## 2 Synthetic cells as a continuum of complexity

Synthetic cells are not a single, discrete category, but a continuum of constructs that vary in complexity, from non-replicating biochemical assemblies to genome-containing, potentially self-replicating systems (NASEM, 2026). Advances in nucleic acid synthesis, cell-free systems, and genome design are expanding the diversity of synthetic cells while lowering technical barriers to constructing them (Bundy et al., 2018; Perez et al., 2016; Dondapati et al., 2020).

Synthetic cells that incorporate non-traditional biochemistries, including xenonucleic acids or alternative chirality biomolecules, as well as those that rely on synthetic or redesigned genomes or hybrid architectures, have direct implications for governance (Pinheiro et al., 2012; Hutchison et al., 2016; Adamala et al., 2024; NASEM, 2026). In these cases, sequence-based screening, attribution methods, and organism-centered oversight approaches may fail to identify or interpret relevant risks (Wittman et al., 2025; Millett et al., 2023; Diggans and Leproust, 2019; NASEM, 2018). Risk becomes less tied to identifiable organisms and more closely linked to the design, assembly, and function of underlying components, particularly nucleic acids that may fall outside existing databases or screening frameworks (Schmidt, 2008; Solé, 2016; Appleton et al., 2017).

As a result, synthetic cells blur distinctions between living and non-living systems and challenge assumptions that risk can be inferred from organism identity or known genetic sequences (Metzler and Webster, 2011; Samimian-Darash et al., 2016; Meloni, 2016; Rijssenbeek, 2025; NASEM, 2026). Improved tools are needed to detect and monitor the persistence of systems that contain novel or non-standard components (NASEM, 2026).

These dynamics highlight a central governance challenge. Existing approaches are often calibrated to known categories and systems, while synthetic cells increasingly span a continuum of designs and functions.

## 3 A framework based on design, function, and context

The challenges outlined above motivate a shift from category-based oversight toward evaluation grounded in system design, function, and behavior. The report provides a foundation for this shift through a set of core evaluative questions designed to support decision-making across institutional and regulatory environments (see Box S-1 in NASEM, 2026, *Core Questions Guiding Classification and Risk-Benefit Evaluation* and Appendix D in NASEM, 2026, *Biosecurity, Dual Use and Cross-Domain Oversight Questions for Synthetic Cell*). These questions in in the NASEM, 2026 provide a structured way to guide risk assessment in cases where classification under existing governance frameworks is insufficient. Empirical work on biosafety practice shows that institutional review already depends heavily on interpretation and experience-based reasoning, particularly when systems do not align with established categories, which is likely to be the case for many synthetic cells (Gillum, 2025b; Gillum et al., 2026a; NASEM, 2026). For researchers, biosafety officers, and Institutional Biosafety Committees (IBCs), the report offers a common methodology for evaluating systems whose properties do not map cleanly onto existing oversight mechanisms.

Work in synthetic biology governance has found that risk and benefit often arise from similar system properties, especially in technologies that enable new forms of biological design and deployment (Oye et al., 2014; Trump et al., 2020). In such systems, risk and benefit must be evaluated together, because both emerge from the same underlying features. Benefit-oriented questions complement risk-based evaluation by clarifying the value, distribution, and justification of expected outcomes. Building on the report’s evaluative framework, Table 1 provides a structured set of questions that more explicitly address the assessment of benefits alongside risks.

**TABLE 1:** Benefit-oriented questions to support balanced evaluation.

Question	Why it matters	How it complements risk-focused evaluation
What is this research or application designed to do?	Establishes a clear purpose for the research or application	Grounds evaluation in intended function, not just materials or hazards
What would success look like in practice?	Defines concrete, observable outcomes (e.g., reduced disease, cleaner environments)	Makes benefits measurable, similar to how risks are assessed
How meaningful is the benefit for those affected?	Distinguishes between minor improvements and significant outcomes	Adds depth beyond simply counting how many are affected
Who benefits, how significantly, and are any groups excluded?	Identifies both beneficiaries and gaps in access or impact	Complements risk discussions about who may be exposed or affected
Does this research or application address a need that is currently underserved or overlooked?	Highlights work that fills important gaps, even for smaller or less visible groups	Prevents benefits from being judged only by majority impact
Who has a voice in determining whether this research or application is valuable?	Recognizes that different communities may define value differently	Supports transparency and public engagement in decision-making
What makes this research or application better than existing options?	Identifies whether it offers meaningful improvement (e.g., effectiveness, accessibility)	Avoids accepting risk for marginal gains
Under what conditions does the research or application deliver the benefit?	Specifies where and how the system must operate to be effective	Aligns benefit assessment with real-world use conditions
How long does the benefit last?	Distinguishes between temporary and sustained effects	Mirrors risk considerations such as persistence and exposure duration
Does the research or application interact with living systems to achieve its benefit?	Identifies biological pathways involved in producing the outcome	Links directly to the same mechanisms considered in risk assessment
Could the same feature that enables benefit also introduce risk?	Makes tradeoffs visible (e.g., persistence enabling both benefit and exposure)	Integrates benefit and risk into a single line of reasoning
What evidence supports the expected benefit?	Requires data, prior work, or a clear scientific basis	Applies the same standard of evidence used in evaluating risk
What are the key uncertainties affecting whether the benefit will be realized?	Identifies limits in current knowledge	Aligns with the emphasis on uncertainty alongside risk and benefit
How would we know if the benefit is actually occurring?	Defines how outcomes will be monitored or measured	Parallels monitoring used to assess safety and compliance
What happens if the benefit is smaller than expected or not achieved?	Considers realistic outcomes and opportunity costs	Adds balance by addressing failure of value
Are the benefits broadly shared or concentrated?	Distinguishes between public value and more limited gains	Introduces fairness alongside risk distribution

These questions may be used by IBCs, research institutions, biosafety officers, policymakers, and the public to assess whether the expected benefits of research are clear, meaningful, and justified.

### 3.1 Core logic of the framework

The report’s framework operates through two complementary layers of evaluation (NASEM, 2026). The first focuses on basic characteristics of the system, particularly whether it contains genetic material and whether it is capable of replication. These features are treated as intrinsic properties of the system and serve as initial entry points for evaluation.

In frameworks such as the National Institutes of Health (NIH) *Guidelines for Research Involving Recombinant or Synthetic Nucleic Acid Molecules* (hereafter “NIH Guidelines”), risk assessment is structured differently. Oversight is organized around predefined categories of experiments, with risk inferred from the source and identity of genetic sequences and their relationship to known biological systems. For example, experiments are flagged based on whether they involve sequences derived from pathogenic organisms, genes encoding toxins, viral elements, or whole-organism modifications in plants and animals. These determinations are tied to the Risk Group of the parent organism, the host–vector system, and the experimental category, and are used to assign containment levels and review requirements (NIH, 2024). In practice, this approach relies heavily on sequence identity and analogy to known organisms and constructs.

**FIGURE 1:**
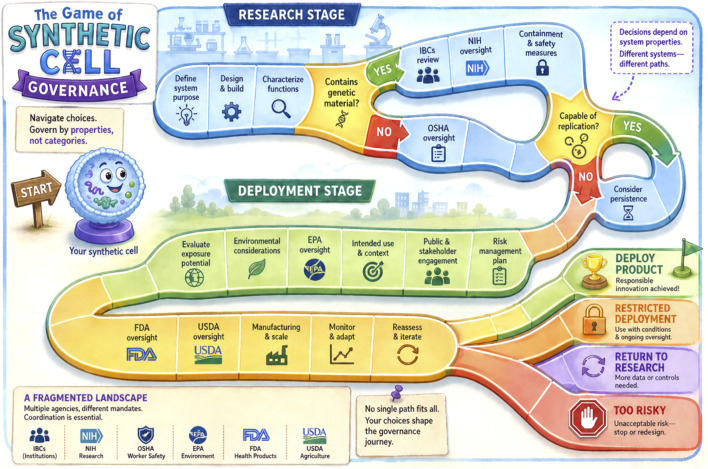
Notional pathways of synthetic cell governance across the research-to-deployment lifecycle. A board game–inspired pathway highlights how synthetic cell research and development navigates key decision points based on system properties (e.g., presence of genetic material, capacity for replication) and transitions across distributed oversight contexts, including research-stage review (e.g., IBCs, NIH, OSHA) and deployment-stage regulation (e.g., EPA, FDA, USDA). This figure is a conceptual representation and does not correspond to any specific regulatory process—the reader is invited to consider the non-idealities implicit in the pathways shown. This figure was conceptualized by the authors and generated using AI tools (ChatGPT, based on GPT-5.3).

The report takes a different approach. It does not use sequence origin or similarity as the primary basis for assigning risk. Instead, it evaluates systems based on their properties, including whether they contain genetic material and whether they can replicate, and then extends this assessment to how the system is expected to behave in a given setting. These properties are not used to place systems into predefined categories, but to support a broader, property- and context-based evaluation of factors such as persistence, interaction with living systems, and deployment environment. This shift allows the framework to be applied to systems that do not map cleanly onto existing organism- or sequence-based categories. See Figure 1 for a notional representation of the decision pathways for governance.

The second layer of the report’s framework evaluates how systems function outside controlled settings. This includes intended use, deployment environment, persistence, interaction with living systems, potential for evolution, exposure pathways, scalability, and the introduction of novel components, as well as considerations of accessibility and misuse potential (NASEM, 2026). This layer shifts evaluation from what a system *is* to how it *behaves* in real-world contexts.

This approach aligns with broader efforts in biosafety and biosecurity to move beyond checklist-, sequence- and category-based compliance toward structured, scenario-informed evaluation of system behavior and potential impact (Dettmann et al., 2022; NASEM, 2026). The report applies this framework to eight cases spanning a wide range of designs and applications, illustrating how reliance on organism-based classification, sequence similarity, and predefined experimental categories can break down when systems do not map cleanly onto existing biological frameworks (see Chapter 5, *Cases Across the Continuum* in NASEM, 2026).

### 3.2 Implications for consistency and decision-making

Several features of this evaluative framework are worth highlighting. First, the 11 questions in Box S-1 of the report can be applied across settings, from laboratory research to environmental deployment and across therapeutic, agricultural, industrial, or environmental applications (NASEM, 2026). This will promote greater consistency across institutions and reduce the likelihood that similar systems will be evaluated differently. Such consistency matters because studies of biosafety and biosecurity implementation have documented substantial variation in how institutions interpret and apply oversight requirements, reflecting differences in expertise, resources, and local risk cultures (Gillum, 2025b; Chamberlain et al., 2009).

Second, the questions in the report are iterative. Some cannot be answered definitively at early stages of research. Evolvability, for example, depends on replication fidelity, mutation rates, selection pressures, and environmental conditions. Governance approaches for emerging technologies increasingly emphasize revisiting assumptions as systems move toward application (NASEM, 2018; Evans et al., 2020; Wynne, 1992).

Third, the questions are applicable across the research and development lifecycle rather than only at the point of formal biosafety review (Gillum, 2025a). Early in development, they can guide experimental design by helping researchers anticipate how persistence, interaction, scalability, detectability, and deployment conditions may shape downstream use. Because many risk-relevant properties are established during design, using a shared evaluative framework across stages can reduce the likelihood that important concerns are introduced too late.

The risk-assessment questions included in the report, as well as the benefit-assessment questions provided in Table 1, could be incorporated into IBC protocols, pre-review materials for grants, and project summaries, and used to inform earlier-stage design decisions before formal review is required. For IBCs, there is a strong case for adopting a baseline set of such questions as part of routine review. Current practices vary across institutions, and IBCs frequently operate at the boundary between formal regulatory requirements and practical decision-making. Emerging technologies expose gaps between policy categories and the systems under review (Burnette and Connell, 2016). A shared baseline would not eliminate variation, but it would provide a more consistent starting point for evaluation and clearer communication across institutions.

## 4 Governance gaps across institutions and development stages

### 4.1 Distributed oversight and misalignment in practice

Considerations related to potential misuse actors are not absent from current biosecurity systems. They are addressed through a distributed set of governance mechanisms across the research and development lifecycle, including institutional biosafety and biosecurity programs, research governance processes, personnel oversight, and nucleic acid synthesis screening (Perkins et al., 2019; Kane and Parker, 2024; Gómez-Tatay and Hernández-Andreu, 2020; Gillum et al., 2026). Governance of dual-use research and emerging biotechnology requires balancing scientific innovation with safety and security obligations (Selgelid, 2009; Koblentz, 2014; U.S. Department of Agriculture, 2020; Epstein, 2025). The challenge addressed here is different: how institutions identify, evaluate, and revisit questions related to system properties, functional characteristics, deployment context, and misuse potential as technologies evolve. This shifts attention from oversight triggers alone to the ongoing assessment of system behavior, intended use, and potential consequences across the research lifecycle.

Oversight of synthetic cell systems operates across a distributed governance ecosystem, with responsibilities divided among institutional review bodies and federal agencies (NASEM, 2026). Early-stage research is typically reviewed through institutional biosafety processes, particularly IBCs operating under the NIH Guidelines, while later-stage applications engage regulatory authorities based on product characteristics and intended use (NIH, 2024; NASEM, 2017; U.S. Department of Agriculture, U.S. Environmental Protection Agency, & U.S. Food and Drug Administration, 2023). This structure reflects not an absence of oversight, but its fragmented distribution across institutions and stages of development (NASEM, 2026; NASEM, 2017).

A key implication is misalignment between when risk-relevant features emerge and when formal oversight is triggered. Synthetic cell systems may acquire properties related to persistence, interaction, or deployment context well before they fit within regulatory categories, so governance questions often arise earlier in the research lifecycle than formal review. The report characterizes this as a timing challenge rather than a gap in oversight (NASEM, 2026). These dynamics reflect a broader feature of U.S. biotechnology governance, which is organized around existing statutory authorities that engage at different stages rather than a continuous framework (NASEM, 2017; Executive Office of the President, 2016).

### 4.2 Limits of categorical triggers and implications for governance

Many oversight frameworks rely on categorical triggers, while synthetic cell systems are better defined by their design, function, and intended use (NASEM, 2026). Although organism type, genome sequence, and product category provide entry points for oversight, they do not always correspond to how systems behave or where risks and benefits arise (NASEM, 2026; NASEM, 2017; Executive Office of the President, 2016). As a result, systems with similar functional properties may be evaluated through different pathways depending on classification or timing (Maxon, 2023). For example, a non-replicating synthetic cell designed for environmental sensing may be treated differently depending on whether it contains nucleic acids, even when persistence and environmental interactions are similar.

In research settings, the NIH *Guidelines* provide a central biosafety framework, but their applicability depends on triggers such as the presence and characteristics of nucleic acids (NIH, 2024; Gillum et al., 2026b). This creates challenges for systems involving non-standard or engineered nucleic acids, as well as hybrid systems that combine biological and non-biological components (NASEM, 2026). More broadly, trigger-based governance may not capture system behavior, exposure pathways, or functional activity, particularly for systems that span chemical and biological domains (Kuzma and Tanji, 2010; NASEM, 2026).

Similar patterns appear across the governance landscape, where oversight is triggered by product characteristics and intended use rather than system behavior (CDC and NIH, 2020; U.S. Department of Agriculture, U.S. Environmental Protection Agency, and U.S. Food and Drug Administration, 2023). As a result, similar systems may fall under different authorities depending on features such as genetic content or replication capacity, contributing to fragmentation (NASEM, 2026).

### 4.3 Implications for oversight in practice

These dynamics are particularly evident when systems are evaluated in terms of intended use. As illustrated in the report’s case studies, a non-replicating synthetic cell designed for environmental remediation may require persistence to function effectively, even though non-replication is often treated as a proxy for lower risk (NASEM, 2026). Therapeutic synthetic cells intended for *in vivo* use raise questions about persistence, reversibility, and host interaction, while synthetic cells for biomanufacturing may offer containment advantages but still present challenges related to monitoring and performance at scale. Across these examples, classification alone is not a sufficient guide to oversight, because risk and benefit emerge from the interaction of system properties and context (NASEM, 2026).

The U.S. approach to biotechnology oversight relies on statutes applied by multiple agencies based on product characteristics and intended use rather than a unified framework (U.S. Department of Agriculture, U.S. Environmental Protection Agency, and U.S. Food and Drug Administration, 2023). While this provides flexibility, it also means oversight becomes clearer later in development, even as key questions arise earlier. A central issue is therefore how well timing and scope align with the trajectory of emerging systems (NASEM, 2026).

Synthetic cell research highlights limits in how existing frameworks align with systems that are hybrid, application-driven, and difficult to categorize. The report points toward improved alignment through coordination, information sharing, and shared evaluative frameworks focused on system properties, functions, and contexts of use (NASEM, 2026). Integrating these approaches into funding review, institutional oversight, and interagency coordination could improve consistency without requiring entirely new regulatory structures.

## 5 Education, training, and public engagement

Operational governance requires stronger capacity to evaluate systems whose risks and benefits emerge from the same design choices and operating conditions (Jasanoff, 2004; Oye et al., 2014). In practice, technical capability, intended benefit, uncertainty, and oversight are considered together, but they are often treated separately in training and governance.

Synthetic cell research highlights the limitations of this separation. Scientific training typically emphasizes system design and performance optimization (AAAS, 2011; NRC, 2009), while biosafety and biosecurity frameworks focus on hazard classification and compliance (NRC, 2004b; Koblentz, 2010). Because synthetic cell risks and benefits depend on how design features, uses, and uncertainties interact, governance requires integrating these perspectives.

Case-based approaches provide a practical way to do so. The report uses cases to link system properties to potential outcomes and oversight considerations, making governance questions concrete and teachable. Questions about replication, persistence, interaction, intended use, and misuse potential are integral to understanding how systems function and how they may behave outside controlled environments. Embedding these approaches into training would help align scientific reasoning with governance expectations and support more consistent evaluation across institutions.

This is particularly important under conditions of uncertainty. Many synthetic cell systems are at early stages of development, where empirical data are limited and behavior may not be fully predictable. Governance in such settings requires iterative and adaptive approaches that account for limits of prediction and control (Collingridge, 1980; Jasanoff, 2003; Stilgoe et al., 2013). Biosafety and biosecurity must therefore prepare scientists (those generating knowledge) and practitioners (those applying, managing, or overseeing synthetic cell research, development, and governance, including engineers, technicians, industry actors, and regulators) to identify what is known, what remains uncertain, and what evidence is needed to support responsible decisions, consistent with the report’s emphasis on empirical gaps and adaptive oversight.

Effective governance also depends on producing data that reduces uncertainty in areas directly relevant to oversight. This includes experiments that clarify persistence, environmental interaction, detectability, horizontal gene transfer potential, and the performance of monitoring and containment strategies. Such work is especially important for systems involving synthetic nucleic acids, non-standard chemistries, and novel genetic architectures, where conventional assumptions about detection, attribution, and sequence-based risk assessment may not hold (Schmidt, 2008; Solé, 2016). Linking empirical research priorities more explicitly to governance needs would help ensure that evaluation is based on evidence rather than reliance on precedent, analogy, or opinion alone.

Scenario-based training can help build the expertise required for these decisions by using realistic cases drawn from across the synthetic cell continuum to practice applying risk-benefit frameworks, identifying relevant uncertainties, and selecting appropriate oversight and monitoring approaches under conditions of incomplete evidence (Herreid, 2011). The same logic applies to graduate education, where researchers working on synthetic cell development should be prepared to anticipate how their work may be evaluated, how it may be used or misused, and what evidence will be necessary for responsible deployment (Rappert, 2014; Minehata and Shinomiya, 2010).

Programs such as the International Genetically Engineered Machine (iGEM) competition have shown that students can engage meaningfully with biosafety, biosecurity, and ethical considerations when they are embedded in project-based work (Smolke, 2009). Adapting the report’s case studies for earlier education and outreach could support developing habits of reasoning about system behavior, consequences, and use. Public engagement and responsible innovation frameworks promote transparency, reflection, and dialogue where emerging technologies may have broad societal impact (Stilgoe et al., 2013). By grounding abstract governance questions in concrete cases, the report offers a basis for more informed discussion among scientists, policymakers, and the public.

## 6 Evidence gaps and experimental needs

The broad spectrum of possible synthetic cells also highlights the need for a stronger body of evidence for evaluating risks (NASEM, 2026). For example, synthetic genomes enabling novel genetic codes have shown resistance to bacteriophage infection (Zürcher et al., 2025), but it is unknown how this feature could influence those cells’ persistence in the environment or competitiveness with native species that are subject to phage predation. Similarly, inclusion of novel synthetic nucleotides may challenge standard detection methods, making such a synthetic cell difficult to monitor (Hoshika et al., 2019; Pinheiro et al., 2012; Diggans and Leproust, 2019). Thus, the report specifically recommends that U.S. research funders “should invest in systematic studies and data infrastructure to fill empirical gaps supporting synthetic cell risk and benefit assessment” (NASEM, 2026) and provides several priority areas. For example, crucial experiments should test the extent of persistence, survivability, and horizontal gene transfer involving synthetic cells—tested under realistic conditions beyond laboratory or contained manufacturing environments. In addition, the report recommends that means of building unique containment features into synthetic cells should be explored and thoroughly characterized.

## 7 Toward operational governance

Synthetic cells generally do not introduce fundamentally new categories of risk, but they expose limits in governance systems built around static categories and familiar biological assumptions (NASEM, 2026). These limitations are especially evident for synthetic nucleic acid systems, where non-canonical or engineered components challenge sequence-based approaches to screening, attribution, and detection. In such cases, risk cannot be reliably inferred from recognizable sequences or established organismal categories alone.

Building on the report’s framework, this analysis shows how governance challenges arise from the interaction of categorical triggers, institutional boundaries, and the timing of oversight across the research and development lifecycle. Oversight is typically structured around classification-based entry points, while risk-relevant properties, such as persistence, interaction, and intended use, emerge earlier and evolve over time, creating misalignment between evaluation and real-world behavior.

These observations also highlight limits in how current risk assessment approaches are applied. Existing frameworks provide important foundations, but do not fully capture how risk and benefit emerge from the same system properties or how these interact with deployment context. The structured questions in Table 1 support more explicit evaluation of benefits alongside risks, helping decision-makers assess both potential harms and the value of expected outcomes.

Operationalizing this approach could involve incorporating core evaluative questions into IBC review materials, funding proposals, and interagency coordination, alongside prioritizing empirical work that reduces uncertainty in persistence, detectability, and environmental interaction. Together, these steps would better align governance with system properties, intended use, and deployment context while building on existing institutional structures.

## Data Availability

The original contributions presented in the study are included in the article, further inquiries can be directed to the corresponding author.
